# Fabry Disease in Women: Genetic Basis, Available Biomarkers, and Clinical Manifestations

**DOI:** 10.3390/genes15010037

**Published:** 2023-12-26

**Authors:** Raafiah Izhar, Margherita Borriello, Antonella La Russa, Rossella Di Paola, Ananya De, Giovambattista Capasso, Diego Ingrosso, Alessandra F. Perna, Mariadelina Simeoni

**Affiliations:** 1 Department of Mental and Physical Health and Preventive Medicine, University of Campania “Luigi Vanvitelli”, 80138 Naples, Italy; rosella.dipaola@unicampania.it (R.D.P.); ananyade150111997@gmail.com (A.D.); 2 Department of Precision Medicine, University of Campania “Luigi Vanvitelli”, 80138 Naples, Italy; margherita.borriello@unicampania.it (M.B.); diego.ingrosso@unicampania.it (D.I.); 3 Department of Sperimental Medical and Surgical Sciences, Magna Graecia University, 88100 Catanzaro, Italy; lrantonella@yahoo.it; 4 Biogem S.c.a.r.l. Research Institute, 83031 Ariano Irpino, Italy; gb.capasso@unicampania.it; 5 Nephrology and Dialysis Unit, Department of Translation Medical Sciences, University of Campania “Luigi Vanvitelli”, 80131 Naples, Italy; alessandra.perna@unicampania.it

**Keywords:** Fabry disease, *GLA* gene, genetic basis, X-chromosome inactivation, clinical manifestations, therapies, quality of life

## Abstract

Fabry Disease (FD) is a rare lysosomal storage disorder caused by mutations in the *GLA* gene on the X chromosome, leading to a deficiency in α-galactosidase A (AGAL) enzyme activity. This leads to the accumulation of glycosphingolipids, primarily globotriaosylceramide (Gb3), in vital organs such as the kidneys, heart, and nervous system. While FD was initially considered predominantly affecting males, recent studies have uncovered that heterozygous Fabry women, carrying a single mutated *GLA* gene, can manifest a wide array of clinical symptoms, challenging the notion of asymptomatic carriers. The mechanisms underlying the diverse clinical manifestations in females remain not fully understood due to X-chromosome inactivation (XCI). XCI also known as “lyonization”, involves the random inactivation of one of the two X chromosomes. This process is considered a potential factor influencing phenotypic variation. This review delves into the complex landscape of FD in women, discussing its genetic basis, the available biomarkers, clinical manifestations, and the potential impact of XCI on disease severity. Additionally, it highlights the challenges faced by heterozygous Fabry women, both in terms of their disease burden and interactions with healthcare professionals. Current treatment options, including enzyme replacement therapy, are discussed, along with the need for healthcare providers to be well-informed about FD in women, ultimately contributing to improved patient care and quality of life.

## 1. Introduction

Fabry disease is a multisystemic, lysosomal storage disease caused by mutations of the *GLA* gene mapping on chromosome X [1,2]. *GLA* is a 14-kb gene located on Xq22.1 that encodes the α-galactosidase A (AGAL) enzyme [3]. It is a polypeptide of 429 amino acids, and the Human Gene Mutation Database lists over 900 variants, of which 69% account for missense mutations [4]. Deficiency in the AGAL enzyme leads to the degradation of glycosphingolipids from cell membranes and causes the accumulation of globotriaosylceramide (Gb3) and other lipid complexes, mostly in vital organs (kidneys, heart, and nervous system) [2]. FD was first described by Johannes Fabry and William Anderson in 1898 [5,6]. 

FD was initially thought to predominantly affect males, having a single uncompensated mutated *GLA* gene. This could result in a severe lack of AGAL activity and lead to the early emergence of symptoms during childhood/adolescence with progressive organ damage and increased death risk [7]. Due to the X-linked inheritance, heterozygous females were considered to be asymptomatic carriers; however, over time, it has become evident that females exhibit a diverse range of clinical symptoms and experience a level of clinical severity often equivalent to that observed in males [8,9]. The reasons behind the diversity in the female phenotype are not yet well understood, although the potential influence of X-chromosome inactivation (XCI) has been postulated. The variation in the phenotype is a result of a process known as “lyonization”, in which one of the two X chromosomes in somatic cells is randomly inactivated [2]. FD occurs in approximately 1 out of 117,000 live births in hemizygotes. However, if we take heterozygotes into account, the occurrence rate can increase to as much as 1 in 58,000 [10].

The classic presentation of FD has been extensively documented in males [7]. Nonetheless, recent studies have indicated that several heterozygous Fabry women undergo substantial disease-related challenges and a diminished quality of life [11]. Clinical features of Fabry disease in women encompass a wide spectrum of symptoms, including angiokeratomas, early strokes, myocardial hypertrophy, arrhythmias, gastrointestinal disorders, and renal involvement. Renal involvement is a prominent aspect of Fabry disease in women due to the mutation in the gene encoding the enzyme AGAL, which causes Gb3 accumulation [12]. This accumulation leads to serious and progressive impairment of renal function, including proteinuria and kidney failure [13,14]. Males are more likely to develop renal manifestations of FD than females [15]. Moreover, the development of renal symptoms in females is delayed compared to males, and the phenotypic heterogeneity of FD in females is corelated with both age and severity of symptoms [16]. In both adult male and female patients with FD, proteinuria and renal dysfunction are frequently detected and are considered the main cause of premature death [14]. Chronic kidney disease (CKD) is an important feature of FD [17], accounting for 0.01% of end-stage renal disease (ESRD) patients enrolled in European and US dialysis registries [18,19].

At present, the primary and fundamental treatment for FD in women is enzyme replacement therapy (ERT). However, alternative therapeutic strategies like chaperone therapy, substrate reduction therapy, and gene therapy are under investigation [20]. In addition, a qualitative study investigating the experiences of heterozygous Fabry women found that they had unfavorable interactions with medical personnel because of their gender, carrier status, and the disease [21], and they may bear a heavy burden of disease and experience a lower quality of life (QoL). To provide appropriate care and support, healthcare providers must be well-informed about FD and the symptoms it presents in women. In this review, we will explore in depth all the peculiar aspects of FD in women. 

## 2. FD Inheritance in Females

**Figure 1:**
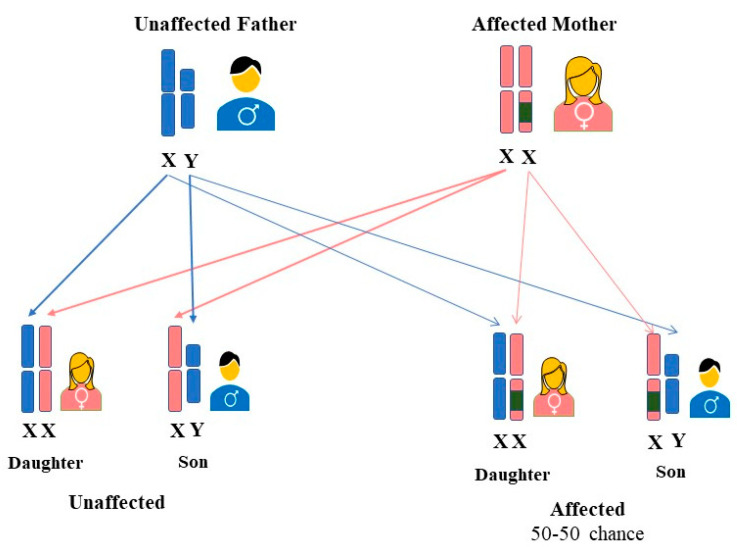
Inheritance pattern of FD in women. A mother with a *GLA* variant on one of her two X chromosomes has a 50% probability of passing FD to each of her offspring, regardless of their gender.

Initially, FD was considered to be an X-linked recessive hereditary disorder that affected primarily hemizygous males [22], whereas females were thought to be the carriers who were either asymptomatic or to have mild symptoms [23]. However, research over the years has shown that most females carrying the mutated gene may exhibit signs and symptoms of the disease [22]. It was observed that heterozygous females could be affected similarly to hemizygous males, and thus FD was postulated to be classified as X-linked [24]. Consequently, FD is now referred to as an “X-linked transmission” disease, and the use of the term “carrier” for women carrying a disease-causing mutation may no longer be correct [25]. The *GLA* gene mutations are typically heterozygous in females. Although a female can have the defective gene on both X chromosomes, the homozygous form is incredibly uncommon [26]. Regardless of sex, an affected woman who is heterozygous for the *GLA* gene mutation has a 50% probability of transferring a faulty gene to both males and females (27). (See Figure 1). 

The random inactivation of one of the two X chromosomes, also known as lyonization, may have an impact on women just as severely as it does on hemizygous men [4]. The activity of AGAL in afflicted women can range from low to normal as a result of lyonization [3]. It has been proposed that XCI may have a potential role in the phenotypic expression of FD in females. In heterozygous females, the disease’s phenotypic diversity is substantially higher than in males, and symptoms typically appear late in life and progress gradually [24]. The rate of disease progression differed among females with the same pathogenic variants, perhaps because of the degree of skewed XCI that favors the mutant *GLA* allele [27]. Studies investigating the prevalence of skewed XCI in females with FD have been published; however, they are ambiguous and contradictory. 

Genotype-phenotype correlations in Fabry disease have been a subject of increasing interest in the scientific community. The Lyon hypothesis, or random X-inactivation, has been proposed as a potential explanation for the phenotypic expression of Fabry disease in heterozygous women [28]. The wide heterogeneity in the natural history of patients with Fabry disease has led to efforts to establish potential associations between genotypes and phenotypic expressions of *GLA* gene [29]. However, reports have indicated that the same mutation can cause different phenotypes in the same family and different mutations can lead to the same phenotype, implying that no clear genotype-phenotype correlation could be identified in Fabry disease [30].

Female heterozygous patients with FD had varying degrees of disease severity, and it became worse as they aged [31]. The phenotypic expression of FD in females is significantly influenced by the degree of XCI [32]. Some investigations identified a correlation between the severity of disease manifestation and the pattern of XCI [33,34,35]. However, others do not corroborate these observations [36,37]. So many authors have dismissed its significance in elucidating and explaining phenotypic variability. The findings of previously published research indicate a correlation between clinical index and X inactivation, with two of four heterozygotes carriers of FD presenting a skewed XCI in favor of the mutant allele and the other two asymptomatic females exhibiting a skewed XCI favoring the wild-type allele [33]. On the contrary, a different study found no variations in the XCI ratio between 28 FD-affected women and healthy women of the same age. Skewed X inactivation was present in only 18% of heterozygous females [36]. Elstein et al. observed similar results among 77 heterozygous females, where only 18.2% had a significantly skewed XCI [37]. These findings do not provide significant evidence for the notion that skewed XCI is related to the severity of disease manifestation among female carriers of the mutant *GLA* gene. 

Classical methods for measuring X-chromosome inactivation (XCI) skewness may not be sufficient to fully explain the manifestations of FD in women. In addition to unbalanced XCI, allele-specific DNA methylation at the promoter of the *GLA* gene can influence the expression levels of the mutated allele, thereby effecting the onset and outcome of FD. DNA methylation is an epigenetic modification that occurs at both X and autosomal genes, primarily at cytosine residues of CpG sites, and can regulate gene expression [38]. Therefore, analyzing DNA methylation at the *GLA* promoter, specifically distinguishing between the mutated and non-mutated alleles, can provide more informative insights. Recent studies have shown that DNA methylation can help in explaining the variability in the clinical phenotype observed in FD [39]. A study by Hossain et al., [40] investigated the linkage between DNA methylation patterns and Fabry disease severity in 36 affected women. They analyzed DNA methylation in peripheral blood and skin fibroblasts using methylation-sensitive restriction enzymes and bisulfite Sanger sequencing. The research identified a distinct correlation among the severity of the phenotype, the accumulation of lysoGb3, and methylation of the normal allele detected by non-digestion with methylation-sensitive restriction enzymes [39].

In a separate investigation, Hossain et al., [41] explored the impact of DNA methylation on the Fabry phenotype. The researcher underscored the significance of unbalanced DNA methylation in the expression of mutated alleles for the *GLA* gene, which can exert a substantial impact on the clinical phenotype. This was demonstrated in a severe case of a heterozygous female Fabry patient who manifested various symptoms, including acroparesthesia, facial dysmorphism, left ventricular hypertrophy, and intellectual disability. 

## 3. LysoGb3 as a Predictor Biomarker for FD Severity

FD, the progressive lysosomal storage disorder, occurs due to a functional defect or absence of AGAL [42,43]. Two neutral glycosphingolipids, globotriaosylceramide (Gb3) and its deacylated form, globotriaosylsphingosine (lyso-Gb3), accumulate in the lysosomes of damaged cells in different organs [43,44]. However, the impact of AGAL substrate accumulation on symptom development has increasingly been demonstrated to involve cellular structures beyond the lysosome. Downstream effects, such as inflammation [45], the production of reactive oxygen species [46], and fibrosis [47], appear to have significant contributions to the development of the disease [48]. Vascular endothelium and smooth muscles, epithelial cells, cardiomyocytes in the left ventricle and atrium, pericytes, valvular fibroblasts, and ganglion cells are the main cellular structures containing lysosomes involved in the accumulation of Gb3 in FD [43,49].

Traditionally, only plasma or urinary Gb3 levels have been employed to determine heterozygosity [50] and to estimate the severity or overall activity of the disease [51,52,53]. However, this method is laborious and time-consuming. Nonetheless, extensive research has revealed that Gb3 is not an ideal disease biomarker because many individuals with late-onset variants exhibit partial maintenance of AGAL enzyme activity; subjective symptoms are less likely to appear in childhood; and FD in females does not exhibit high concentrations of Gb3 in their plasma and urine [54], whose levels in many cases may overlap with those of healthy individuals [55]. This underscores the need for researchers to identify new biomarkers with greater diagnostic sensitivity. Recently, globotriaosylsphingosine (LysoGb3) and its deacylated form have emerged as promising diagnostic tools and have garnered interest as potential biomarkers for FD [54]. Elevated levels of lyso-Gb3 have been observed in the plasma, urine, or dried blood spot (DBS) of individuals with FD, and there is less overlap in lyso-Gb3 levels between patients and control populations [55,56,57,58]. In a recent investigation led by Hiroki Maruyama et al. [59], plasma lyso-Gb3 served as the primary screening marker for identifying classic and late-onset Fabry disease in both male and female patients. The authors emphasized the efficacy of employing plasma lyso-Gb3 screening to identify potential candidates for genetic counseling, thereby revealing unrecognized cases; moreover, this analysis facilitated a decrease in the need for unnecessary genetic testing [60]. 

Plasma lyso-Gb3 has become important in screening and as a diagnostic biomarker for FD to distinguish patients with classical and nonclassical phenotypes and healthy candidates [61,62]. Some studies have attempted to establish cut-off values in order to differentiate individuals with atypical variants from those without FD [63]. Nevertheless, plasma lyso-Gb3 concentrations in these variant cases often exhibit only slight elevations or even remain within the normal range, especially in females [50,64], making it challenging to define a precise cut-off value for identifying heterozygous patients [65]. However, the AGLA activity/lyso-Gb3 ratio in DBS has recently been suggested to be a suitable diagnostic marker in females due to its enhanced detection sensitivity compared to the sole assessment of lyso-Gb3 [66]. In females with FD, the correlation of plasma lyso-Gb3 levels with disease severity and neurological manifestations has been defined, implying that plasma lyso-Gb3 concentration serves as an independent risk factor for left ventricular mass [67]. Other research on patients with both classic and atypical phenotypes reported relationships between plasma lyso-Gb3 concentrations and both left-ventricular mass in females [54] and ventricular hypertrophy in females [68]. Additionally, Rombach et al. proposed using lyso-Gb3 as a screening tool for females with left ventricular hypertrophy [55]. However, not all studies have identified similar correlations [20,54]. According to Sakuraba et al. [69], an increase in plasma lyso-Gb3 levels is linked to renal involvement in Fabry females, but there remains uncertainty regarding the statistical difference in plasma lyso-Gb3 levels between Fabry females who develop renal involvement and those who do not. 

As lyso-Gb3 stimulates the production of secondary mediators, which lead to glomerular injury [70], it was hypothesized that exposure to plasma lyso-Gb3 could be linked to the advancement of renal dysfunction. Regarding urine lyso-Gb3, its concentrations are correlated with proteinuria and albuminuria but not with glomerular filtration rate (GFR), suggesting that it is not a strong predictor of renal function [57]. Nowak et al. [50] conducted a study involving 18 individuals with classic, later-onset, and benign mutations in which they determined enzymatic activities and LysoGb3 levels, in which they found three of 18 females with *GLA*-mutation confirmed FD had normal AGLA activities but significantly increased serum LysoGb3 levels. AGLA activity in FD heterozygotes is frequently borderline to normal due to stochastic X-chromosomal inactivation. These data suggest that this biomarker could improve the initial clinically relevant FD diagnosis, primarily in female patients. Smid et al., examined LysoGb3 levels in 14 non-classical patients and observed that these levels overlapped with normal levels, irrespective of AGLA activities. The authors emphasized the necessity of further study of LysoGb3, especially in later-onset (or so-called non-classical) heterozygotes [63]. By screening patients for lyso-Gb3, it is also possible to establish a connection between high lyso-Gb3 levels and normal AGLA activity in female patients with intronic GVUS (genetic variations of unknown significance). The detection of high plasma lyso-Gb3 levels reinforces the continuation of further testing to establish an accurate diagnosis, enabling the selection of people with a high-risk FD. Nowak and coworkers reported an increase in lyso-Gb3 levels in females with normal enzymatic activity who exhibited significant clinical symptoms of FD [50]. Another study found a link between lyso-Gb3 levels and imaging signs, along with phenotype and genotype [60,71]. Table 1 presents a list of additional biomarkers in FD women. 

However, these biomarkers have certain limitations, such as their inability to yield detectable results in the plasma or urine of women with classical FD [88,89] and individuals with nonclassical FD subtypes [90]. They also fall short in predicting kidney involvement [57] and lack reliability as biomarkers for assessing disease activity and treatment response in heterozygous women and recipients of renal allografts [52]. Numerous metabolomic investigations have demonstrated that there are analogues or isoforms of Gb3 and lyso-Gb3 molecules characterized by differences in the structure of the sphingosine group [91,92,93,94]. The recent, unique application of plasma lyso-Gb3 contains the levels of six analogues. However, it is worth noting that this enhanced precision in measurement did not lead to an improved diagnostic rate in the patient cohort studied by Haran Yogasundaram et al. [68]. Several studies have presented evidence indicating the existence of lyso-Gb3 analogs in both urine and plasma.

Notably, the proportion of urinary analogue excretion in comparison to that of lyso-Gb3 is higher than what has been reported in plasma [73,95,96,97], rendering lyso-Gb3 analogs in urine a promising candidate as a disease marker. It’s important to emphasize that the excretion profile of lyso-Gb3, Gb3, and their analogs might differ based on the underlying genetic change [97,98]. Consequently, incorporating the comprehensive profile of lyso-Gb3 analogs in urine could be crucial for broadening the diagnostic information accessible to patients. The total concentration of lyso-Gb3 plus its analogues in urine is very sensitive and specific for both classical and nonclassical FD diagnosis [88,99]. However, it’s worth noting that gender significantly influences the urinary levels of lyso-Gb3 and its analogues in pediatric FD [88], with females displaying lower urinary concentrations of all these biomarkers [88,97,98,99]. Notably, distinct forms of lyso-Gb3 and related analogs, including lyso-Gb3 [+16 Da], lyso-Gb3 [+34 Da], lyso-Gb3 [−2 Da], lyso-Gb3 [+14 Da], and lyso-Gb3 [+50 Da], have been identified in the urine of females with classical and nonclassical phenotypes [88]. These findings underscore the importance of investigating lyso-Gb3 analogues to improve the diagnosis, especially for individuals carrying the genotype associated with a late-onset cardiac variant [97]. However, due to the considerable variability in lyso-Gb3 and its analogues detected in urine among different individuals, further research involving a larger patient population is necessary to establish their clinical value [95]. A recent study has demonstrated the impact of enzyme replacement therapy (ERT) on the concentrations of various analogues of LysoGb3 in FD patients’ urine. The results of ERT showed a reduction in the levels of Gb3, LysoGb3, and related analogues in all FD patients. Specifically, in adult female FD patients who underwent ERT, there was a decrease in urinary levels of LysoGb3, LysoGb3 (−28), LysoGb3 (−2), and Gb3 when compared to untreated females. It is worth noting that only two pediatric female FD patients received ERT, but even in their case, ERT treatment resulted in a decrease in urine levels of Gb3, LysoGb3, LysoGb3 (−28), and LysoGb3 (−2) when compared to untreated pediatric patients [97,99]. Investigations of the different plasmatic lyso-Gb3 analogues could offer deeper insights into the tissue of origin of the Gb3 from which they derive. The isoforms and analogs of Gb3 and lyso-Gb3 seem to hold promising methods for detecting unusual variations in female Fabry patients. Nonetheless, additional research is required to establish their diagnostic utility [95].

## 4. FD Symptoms in Women

FD is a multisystem disorder that starts with cellular dysfunction, progresses over several years, and eventually causes organ functional impairment [100]. The classic type of FD manifests in childhood or adolescence and is characterized by angiokeratomas and corneal degeneration caused by endothelial dysfunction [101,102]. There is a substantial intra- and interfamilial variance in age of onset, clinical features, and clinical course. The typical clinical manifestation of FD in males has received extensive research [2]. In the past, it was believed that FD mostly affected men, while women were thought to be asymptomatic carriers. However, over the past decade, there has been a significant shift in the understanding of FD manifestation in females. Recent studies have conclusively demonstrated that the condition is not restricted to male manifestations only [103,104]. Recent studies have recognized that a significant proportion of female FD heterozygotes experience complications, albeit typically in an attenuated form compared to male FD [105]. The presentation of the disease in women varies widely, with clinical phenotypes ranging from asymptomatic individuals to women presenting severity comparable to classically affected men. This variability extends not only to the intensity of symptoms but also to the range of affected tissues and the onset of symptoms. Notably, the onset of initial symptoms and complications in adulthood tends to occur later in females compared to males [11].

**Figure 2:**
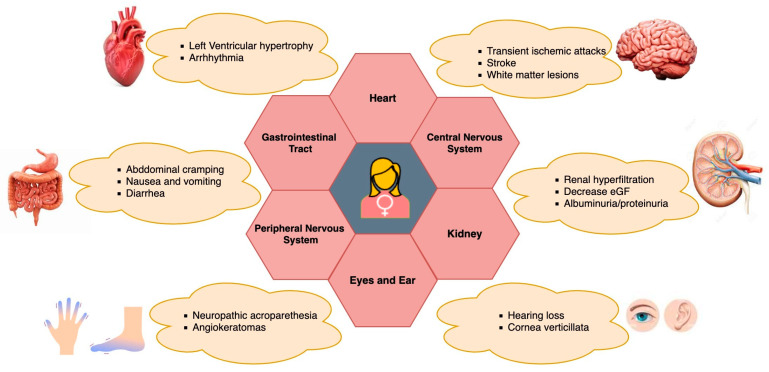
Clinical manifestations of FD in female heterozygotes and its multiorgan involvement.

The main initial clinical manifestations in female heterozygous FD individuals encompass renal, cardiac, and cerebrovascular symptoms, along with gastroenterological disturbances, corneal opacities, and angiokeratoma [104,105,106]. Other symptoms in female FD patients depend on the involvement of the gastrointestinal system, often abdominal pain, diarrhea, or constipation [107] (See Figure 2). Apart from pain, exhaustion, exercise intolerance, and a decreased intake of oxygen are frequently experienced by heterozygous Fabry women [11]. FD appears to be an interesting genetically determined model of chronic kidney disease, chronic neurodegenerative disease, and chronic heart failure (CHF) to investigate the mechanisms of progression of these common diseases [108,109]. Apart from other clinical manifestations, a retrospective analysis of pregnancies among women with FD revealed an increased prevalence of specific pregnancy complications when compared to the broader population of pregnant women. In a study led by Holmes et al., Fabry-affected women completed a survey, revealing that certain Fabry-related symptoms and characteristics, such as gastrointestinal problems, acroparesthesias, proteinuria, headaches, and postpartum depression, could exacerbate during pregnancy. However, there were no documented life-threatening consequences associated with these complications [110]. Specific concerns related to females with FD during pregnancy involve the potential impact of microvascular disease, which may elevate the risk of clotting and exacerbate renal function [9,27]. Also, Gb3 storage has been noted in both maternal and fetal placental tissues, heightening the susceptibility to constrictions in placental blood vessels [111,112,113]. Additionally, there are apprehensions regarding the condition of an affected fetus, leading to pregnancy complications attributed to abnormal Gb3 storage [113]. Concomitant conditions, such as preeclampsia, gestational diabetes, hypertension, and maternal age at delivery, may further complicate pregnancy in women with pre-existing FD [114]. In line with the prevailing Fabry disease guidelines, the survey reinforces the suggestion that pregnant women with FD should undergo assessment and, in certain instances, be monitored during pregnancy by a maternal-fetal specialist, in addition to receiving standard prenatal care [110]. However, further research is still needed to fully understand the specific effects and implications of FD on pregnancy health and outcomes for affected women.

Regarding infertility, a study by Laney et al. [115] revealed that among 197 females with Fabry disease (FD), 40 encountered infertility issues, and 31 out of 225 females sought an infertility assessment to address specific concerns related to infertility. The prevalence of infertility in females with FD surpasses the general population rate, where 12% of women aged 25–44 reported ever encountering infertility issues. However, it is comparable to the number of females in the general population undergoing infertility evaluations [116,117,118]. Out of the 242 females with FD, 30 (12.4%) utilized infertility services in attempts to conceive, employing methods such as donor eggs, in vitro fertilization, and hormonal treatments to enhance ovulation. It is noteworthy that some females opted for Pre-implantation Genetic Screening (PGS) for FD and donor eggs, not due to infertility but to prevent the transmission of FD [115]. Regarding menstrual pain, there is limited specific research and no direct evidence to suggest that menstrual pain is increased in FD women. In a study by Bouwman et al. [119], no substantial difference in the prevalence of premenstrual symptoms, menstrual symptoms, or menarche was observed between females with Fabry disease and the control group. Nevertheless, a higher incidence of reported loss of libido was noted among Fabry disease females compared to the controls. It is important to note that loss of libido may be influenced by the presence of a chronic medical condition [120] and may not necessarily be Fabry-specific. In conclusion, Fabry disease can have implications for pregnancy, infertility, and potentially menstrual health in affected women. However, further research is needed to fully understand the specific effects and implications of FD on these aspects of women’s health.

Renal impairment is one of the prominent features of FD, in which initial indexes include impaired GFR, proteinuria, and tubular derangements [121]. In male hemizygous patients, renal failure is a prominent cause of premature death [122]. In comparison, only 1–2% of females require dialysis or a kidney transplant due to the significantly lower incidence of renal failure in females that leads to end-stage renal disease (ERSD) [28]. A case report of a 26-year-old female patient with FD was published by Kriegsmann and colleagues, where histological, immunohistochemical, and electron microscopic analysis of a kidney biopsy revealed glomerulonephritis, extracapillary proliferative (crescentic), and granulomatous interstitial nephritis [123]. Fabry nephropathy in females is more common and varied than previously believed, according to a thorough review of renal data from 1262 untreated patients (54% of whom were female) included in the Fabry Registry [106]. At the same mean age as males, a considerable number of females had developed moderate to severe renal dysfunction [124]. The median age of occurrence for females with renal progression has been observed to be 38 years [125].

In accordance with MacDermot et al. [105] and Whybra et al. [126], women with FD frequently exhibit cardiac involvement and evidence of structural cardiac damage, especially left ventricular hypertrophy (LVH) cardiomyopathy and mitral valve insufficiency. This constitutes an important contributor to morbidity and mortality [42]. The majority of female Fabry cardiomyopathy patients possess additional characteristics, such as replacement fibrosis and compromised regional myocardial function [127]. Globotriaosylceramide accumulates in the heart when there is cardiac involvement in FD-affected women, leading to cardiac hypertrophy that could deteriorate and result in diastolic filling impairment and contractility impairment. Atrioventricular conduction abnormalities, arrhythmias, coronary insufficiency, and valvular involvement are other potential symptoms of cardiac involvement [42]. Cardiovascular events manifested at a median age of 47 years, which is higher than the average age of men (41 years) [124]. A range of symptoms, such as peripheral neuropathy, pain, autonomic dysfunction, and central nervous system manifestations, have been associated with neurological involvement in FD-affected women. As the disease progresses, both the peripheral and autonomic nerve systems are affected, resulting in paresthesia and neuropathic pain [8]. In females with FD, stroke and transient ischemic attacks (TIAs) are quite prevalent, and they are likely to be brought on by a combination of endothelial dysfunction and aberrant vascular function regulation [27]. The initial screening study for adolescents with cryptogenic stroke was published in 2005 and indicated a significant number of patients comprising a diagnosis, with 2.4% being females [127]. In a published study, it was found that female patients developed cerebrovascular complications from stroke and TIAs more frequently than male patients (27% compared with 12%) [28]. Such problems have been reported in 5–27% of heterozygous females [8,9,10]. About one-third of female patients with neurovascular conditions reported additional symptoms such as vertigo, tinnitus, hearing loss, and hyperhidrosis. Galanos et al. hypothesized that anhidrosis in females may be an indication of later serious renal disease [128]. The cerebrovascular symptoms in females appear to start at an average age of 43 years, thus later than in males (age, 38 years) [124].

## 5. FD Diagnosis in Women Is a Challenging Process

The diagnosis of FD is often made late, especially in pediatric patients, because of the initial non-specificity of presenting symptoms and the lack of widespread awareness among clinicians [32,43,129], which makes the diagnosis delayed and complicated. According to recent studies, there may be up to 15-year diagnosis delays. Because of delayed diagnosis, the damage caused by FD is irreversible [129]. Clinical, radiological, and laboratory analyses play a crucial role in determining the onset and progression of a disease. Nevertheless, establishing the effectiveness of a clinical treatment can be challenging at times, given the substantial variability observed among patients [109]. Nonetheless, biomarkers are crucial for disease and treatment monitoring. In order to diagnose FD in index patients, the first laboratory test is typically an assessment of AGAL activity; if a known family *GLA* mutation is present, a basic DNA analysis is advised. Although a variety of biological samples can be employed for the analysis of AGAL activity, plasma, leukocytes, fibroblasts, and a dried blood spot (DBS) are most frequently used. DBS tests, a test for leukocytes, are frequently used for screening purposes [130].

**Figure 3:**
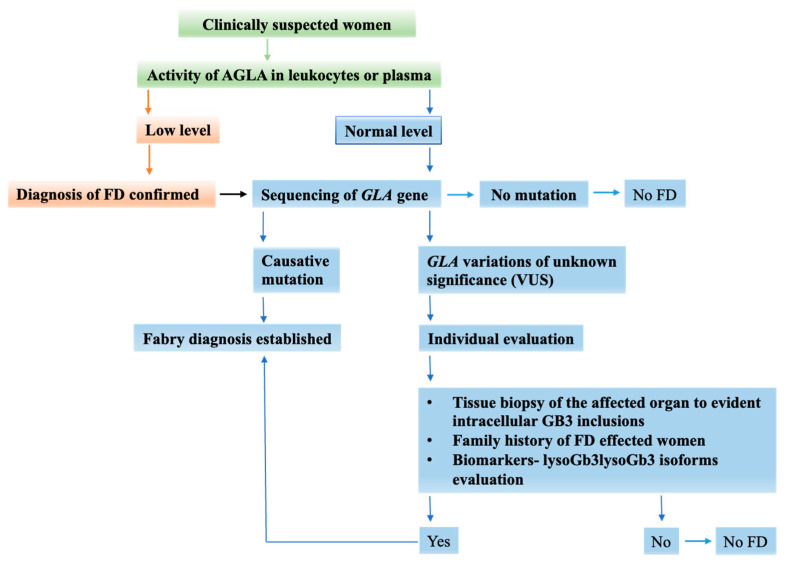
Diagnostic Algorithm for diagnosing women effected or suspected of having Fabry disease.

As FD is an X-linked disorder, homozygotic males exhibiting low AGAL activity can be quickly and easily diagnosed and verified to have the condition. This can be performed by utilizing a synthetic water-soluble substrate, 4-methylumbelliferil-α-d-galactopyranoside [131] to evaluate AGAL activity in plasma [132,133], serum, urine, and leukocytes [132,134]. Screening is conducted using DBS. DBS analysis results that are below the normal range frequently necessitate a confirmation test on leukocytes [106,130]. Sequencing of the *GLA* gene is essential for molecular confirmation in the case of a positive or dubious result [45]. Although it is not necessary for diagnosis, it may offer significant insights for prognosis, therapy, and genetic counseling. AGAL levels in the case of suspected FD heterozygous females can range from deficient to normal levels and are typically inconclusive for diagnosis because of random inactivation of the X chromosome in the cells of the sample [130]. The measurement of α-galactosidase A (AGAL) activity is not a reliable diagnostic criterion for Fabry disease in females. This is due to the varied amounts of AGAL observed in heterozygotes, causing overlap with enzyme levels found in healthy controls [135]. Heterozygous females can manifest a range of illness presentations [106]. In those circumstances, identifying *GLA* pathogenic variants is required to define a female patient’s diagnosis and carrier status utilizing molecular analysis [130,136]. Nonetheless, false-negative outcomes are also prevalent in female patients and some male variants [62,137]. Figure 3 presents the algorithm used to diagnose the suspected female FD individuals.

The gold standard for FD diagnosis in female patients is genetic analysis for mutations in the *GLA* gene [129,130]. The *GLA* gene’s coding exons (which also include the promoter and flanking intronic regions) are now known as one of the best targets for the molecular diagnosis of FD [138,139]. Sequencing the gene’s coding region reveals the pathogenic mutation in more than 97% of patients [44,62,135]. As a result of advancements in high-throughput NGS, genetic panels incorporating the *GLA* gene may now be used to screen high-risk patients, resulting in the detection of multiple *GLA* variations of unknown significance (VUS) [140,141]. If the molecular analysis discovers a variation of unknown significance, supplementary clinical and biochemical tests are required. A slit-lamp examination is recommended to check for the presence of cornea verticillata, which occurs in approximately two-thirds of females [142]. However, in some cases, gene sequencing may fail to detect significant deletions or duplications, resulting in the failure of the technique. It should be noted that false-negative results are frequently observed in female patients and certain male variations. In fact, when it comes to heterozygous females, targeted sequencing methods may fail to identify large gene rearrangements and cryptic splice site mutations [143,144].

It’s worth considering that the *GLA* gene contains a significant number of Alu repeat elements, with approximately one Alu repeat per kilobase [145]. In cases of heterozygous FD patients, Alu-Alu recombination events may not be detected through sequencing, leading to negative results [45]. The majority of pathogenic variants found in individuals with FD are unique to specific families, referred to as private mutations. Mutations that occur in more than one family tend to be located in CpG dinucleotides [146,147]. DNA methylation has been shown to impact the clinical phenotype of FD in women. It can influence the severity and manifestation of symptoms, and it may interact with other factors, such as XCI patterns, to determine the variability observed in FD [39]. It is notable that the absence of a family history of the disease does not necessarily rule out FD, as new pathogenic variants that arise spontaneously (de novo mutations) can also occur [124]. For individuals with FD, it is crucial to conduct pedigree analysis and seek genetic counseling [148]. Family members may also need genetic testing to determine their risk of inheriting the condition. To identify a specific genetic mutation within a patient’s family, various methods can be employed, such as restriction enzymes, probes designed for specific alleles, or preferably DNA sequencing of the gene segment containing the mutation [149]. The efficiency of mutation detection has been markedly improved with the advent of rapid DNA sequencing tests. Recent research has raised growing concerns about the potential influence of specific single nucleotide polymorphisms (SNPs) located in the noncoding regions of the *GLA* gene [150,151] that could be considered for diagnosis. However, due to limited data and the absence of established guidelines, the clinical significance of these SNPs remains unclear, making it necessary to make therapy decisions on a case-by-case basis. In contrast, variants occurring in the coding regions of the gene directly impact the sequence of the enzyme peptide and, consequently, may also affect its structure and function [152].

In the diagnosis of FD females, particularly those with unfavorably skewed X-inactivation, enzyme activity assays are typically uninformative. Consequently, a quest for a new biomarker emerged in the diagnosis and monitoring of the disease. The solution identified was the measurement of the product of Gb3 degradation, lysoGb3 [129]. Elevated concentrations of Gb3 and lysoGb3 in blood plasma and urine can be utilized to confirm the diagnosis [64,153]. Lyso-Gb3 is similar to Gb3, only lacking the fatty acid chain [54]. Levels of plasma lyso-Gb3 have been correlated with disease severity [130]. Recently, the biochemical role of plasma lyso-Gb3 in diagnosis has gained significant relevance as elevated levels have been observed in both males and females [65]. A high level of lysoGb3 was discovered in one of the published studies in women with normal AGAL activity who subsequently presented a clinically relevant manifestation of the disease, exhibiting advantages over the direct assessment of Gb3. Furthermore, it was shown that ERT causes a decrease in lysoGb3 levels. LysoGb3 is therefore considered a more reliable marker of disease activity. However, analysis of long-term data is still required [50]. Sensitive LC-MS techniques for this have been developed [72]. The detection of elevated lysoGb3 levels is very helpful to confirm the FD diagnosis in females [79]. However, many women continue to show normal Lyso-GB3 levels despite a florid phenotype. Although LysoGb3 as a diagnostic marker for FD has been a topic of debate, ongoing efforts are being made to develop more accessible measurement methods for it. Notably, various LysoGb3 analogues or isoforms, including Lyso-Gb3 (−28), Lyso-Gb3 (−12), Lyso-Gb3 (−2), Lyso-Gb3 (+14), Lyso-Gb3 (+16), Lyso-Gb3 (+18), Lyso-Gb3 (+34), and Lyso-Gb3 (+50), have been found to be excreted at increased levels in the urine of FD patients in comparison to the overall level of total LysoGb3. However, some of these isoforms showed reductions post-ERT treatment. Altogether, these findings imply that measuring LysoGb3 isoforms offers a more informative assessment of disease status and treatment response than relying solely on Gb3/LysoGb3 levels [80].

In order to enhance the effectiveness of diagnosing FD in females, a novel biochemical criterion, the AGLA/LysoGb3 ratio, has been suggested. This involves utilizing DBS as a test sample and employing HPLC-MS/MS analysis to measure LysoGb3. Notably, a cut-off value of 2.5 for the α-Gal A/LysoGb3 ratio demonstrated 100% sensitivity and specificity in distinguishing 35 female patients from a control group of 140 individuals. Interestingly, LysoGb3 levels fell within the normal range in 25.7% of females, and the activity of α-Gal A was normal in 91.4% of females with pathogenic *GLA* gene mutations and a family history of classic FD [66]. Furthermore, examining biopsies and confirming the presence of deposits of Gb3/Lyso-Gb3 and its isoforms could provide valuable support for the diagnosis [89,154].

Other options for monitoring FD patients have recently been proposed. MicroRNAs (miRNA, miR) are small RNA molecules that have lately been studied in many illness contexts, and they have been reported to play an important role in FD. There have been a few small investigations into the expression of certain miRNAs in FD. Certain miRNA species, including miR-29 and miR-200, have been linked to renal fibrosis before the onset of severe albuminuria [155,156]. Some serum miRNAs, such as miR-1307-5p, miR-21-5p, miR-152-5p, and miR-26a-5p, have been found to be considerably down-regulated in FD patients by a recent study [87]. In this study, there are both male and female patients with a spectrum of symptoms, suggesting that there could be several sources of circulating miRNAs that come from various cell types and organs. Future research is required to increase the specificity of the findings and to better understand the role of miRNA patterns in the underlying processes of therapeutic benefits. These studies should include appropriate healthy controls [65]. Further validation through larger cohort studies is required to establish the clinical utility of miRNA profiles as biomarkers in FD patients.

## 6. Disease Management

Prior to 2001, there were only symptomatic-based treatments available for FD [157]. These treatments included the use of anti-inflammatory drugs, heart surgery, angiotensin-converting enzyme inhibitors for proteinuria, dialysis, kidney transplantation, and pharmaceutical prevention of ischemic events [43,158,159,160,161,162]. Recent developments in molecular biology and genetic engineering have made it possible for researchers to develop therapies for FD [40]. The objective of currently approved medications such as chaperone therapy and ERT is to decrease intracellular Gb3 accumulation by either replacing the deficient endogenous AGAL or reversing the misfolded endogenous AGAL, which will subsequently enhance trafficking and increase enzymatic activity within the lysosomes [43]. The mainstay of treatment for FD has been ERT [20]. In 2001, two treatment options for ERT in FD, agalsidase alfa (Replagal, Shire HGT) and agalsidase β (Fabrazyme, Genzyme), were introduced in the majority of European countries. The products were recommended to be administered at dosages of 0.2 mg/kg and 1 mg/kg, respectively, every two weeks [43]. Since then, these treatments have also been made accessible in other nations. These two products are formulated using enzymes that are created through gene activation technology and originate from human fibroblasts and the CHO cell line, respectively. The introduction of ERT has given researchers the first chance to address the FD’s underlying enzyme deficit [158,163,164]. Patients with FD who received the enzymes intravenously every two weeks experienced a decrease in both plasma and urine Gb3 levels, as well as a reduction in the accumulation of glycosphingolipids in their capillary endothelial cells, renal glomerular cells, and tubular epithelial cells [13,157]. The timing of treatment commencement becomes more intricate for female heterozygotes with classical FD due to the significant diversity in disease severity and the age at which disease symptoms first appear [159]. The central focus of major clinical trials involving ERT has predominantly revolved around male FD patients. Due to the limited representation of women in these studies, it becomes highly intricate to directly apply the resultant treatment recommendations to the female population. The commencement and advancement of treatment are influenced by the progression of the disease, a factor that varies between female and male individuals with FD [165]. Thus, there is a lack of clarity regarding the optimal timing for commencing ERT and initiating suitable concurrent treatments in females with FD. ERT administered to heterozygous women with FD is safe and efficient [166,167]. Moreover, the use of agalsidase alfa in pregnant women has been administered without causing any adverse impact on either the mother or the newborn [168]. Numerous studies have detailed the stabilization of kidney function in individuals undergoing ERT.

In Fabry patients who have not received any targeted treatment, the organ most prominently impacted is the heart, both in patients with non-classical FD and in females with classical FD. According to several studies, ERT in women initially caused a decrease in heart mass. However, the majority of the included studies were short-term (2–36 months) [139,159]. Agalsidase alfa (Replagal^®^; TKT Europe, 5S AB-Danderyd, Sweden) was administered intravenously to female patients with FD in an open-label, single-center study by Bähner and colleagues to assess its safety, effectiveness, and pharmacokinetics [167]. Agalsidase alfa was demonstrated to be well received, and not a single female patient exhibited the development of antibodies or encountered infusion-related problems. The pharmacokinetic characteristics of agalsidase alfa in female patients closely resembled the pharmacokinetics observed in male patients. Moreover, there was a substantial enhancement in life quality. During the 13–41 weeks of observation, the renal function of these female patients did not decline. It was determined that heterozygous females with FD responded well to ERT with agalsidase alfa [17].

The Fabry Outcome Survey (FOS) patient organ-specific data analysis has shown that ERT with agalsidase alfa improves cardiac structure and function and stabilizes renal function [151,169]. In three clinical trial publications, the outcome of cardiac left ventricular mass (LVM)/left ventricular mass index (LVMi) was presented. All three studies utilized echocardiography to assess LVM. A randomized, multicenter, open-label study, which involved 18 FD women treated for 12 months with different agalsidase alfa regimens (0.2 mg/kg every other week, 0.2 mg/kg weekly, or 0.4 mg/kg weekly), revealed that the use of agalsidase alfa at a dosage of 0.2 mg/kg was linked to a stable LVMi [170]. Another publication reported LVMi outcomes in a cohort of 22 female patients (mean age 44 years) treated with agalsidase β for a median of 36 months, resulting in a significant reduction in LVMi assessed through echocardiography, particularly in patients with left ventricular hypertrophy (LVH) at baseline [171]. Concerning renal involvement in FD disease women, four published studies indicated minimal eGFR loss over time with agalsidase alfa, with changes in eGFR ranging from <1 to −2 mL/min/1.73 m^2^/year [172,173,174,175]. However, for agalsidase β, a research study showed a reduction in eGFR among 62 female patients receiving ERT, although specific statistical details were not provided [176]. Gastrointestinal (GI) outcomes in female patients were reported in two observational studies involving 25 female patients (including pediatric patients) with agalsidase alfa. This study demonstrated a reduction in abdominal pain after 24 months of enzyme replacement therapy, although the incidence of diarrhea had increased in female patients compared to baseline levels at this time point [177]. A 51-year-old woman’s pulmonary symptoms significantly improved clinically, and her lung function partly improved after four years of ERT. CT scans, however, consistently revealed fibrotic changes [178].

In one of the studies, it was discovered that regardless of the recombinant enzyme employed, ERT resulted in stable or decreased plasma lysoGb3 levels in Fabry females [20]. Another study showed that females with classic Fabry had higher plasma lysoGb3 levels before therapy, and ERT resulted in a decrease in lysoGb3 levels [69]. ERT has been proven to improve the prognosis and QoL of treated patients, including women with FD [21].

## 7. Quality of Life and Psychological Aspects

FD is correlated with poor QoL, and this is due not only to the high symptom burden, but also to the life changes involved in therapies such as ERT and hemodialysis [179]. Although heterozygous females with one mutant and one normal copy of the gene can sometimes experience significant symptoms and a decreased QoL, FD has traditionally been assumed to exclusively afflict males [11]. According to published research, women who have FD experience a considerable disease burden, a poor QoL, and significant psychological impacts [11,180]. According to a study by Wang et al., heterozygous Fabry women had multisystemic disease and a lower QoL, along with symptoms like angina, diminished vibration sensitivity, and impaired lung function. Women with FD frequently experience fatigue, exercise intolerance, and symptoms of the renal system [8]. As per a study by Lippe et al., [21], FD symptoms in the central/peripheral neurological, cardiac, and renal systems were far more prevalent than would be expected from a random X-inactivation of the normal allele. A large ratio of FD women who participated in a study by Street et al. [154,181] reported generalized symptoms such as weariness, pain, and gastrointestinal problems. 

Women with FD often experience unpleasant interactions with healthcare professionals, as highlighted in studies by Lippe et al., [21] and Gibas et al., [23]. The expression “Unpleasant interactions with medical professionals” in FD context refers to negative experiences, such as feeling ignored, dismissed, and disbelieved by healthcare providers. This is especially relevant for women due to a triple disadvantage: the rarity of the disease, devalued carrier status, and gender biases. Women with FD may face challenges due to a lack of awareness among healthcare providers, which can lead to late diagnoses and delayed treatment, with potential serious consequences given the progressive nature of FD. Efforts to educate and raise awareness among healthcare professionals are crucial to addressing these challenges and ensuring timely and appropriate care for affected women [21,23].

The physical manifestations of this disease, such as cardiac involvement, can further impact psychological well-being. Women with FD may experience ventricular tachycardia, syncope, and fatigue, which can contribute to psychological distress [182]. As per published research and clinical observations, there is a high risk among individuals with FD of developing neuropsychiatric symptoms, including depression, suicidal tendencies, and neuropsychological deficits [183]. A case series has been published that exhibited severe depression in four female heterozygotes with FD [184]. Along with psychological and psychiatric symptoms, a significant ratio of FD patients may exhibit cerebral ischemia with clinically evident cognitive impairment [185,186]. However, until now, systematic reports addressing neuropsychological deficits in FD patients have been lacking. Individuals with reported neuropsychological deficits also appear to exhibit neurological symptoms or significant cerebral lesions that can be verified through brain imaging techniques [183]. 

The most comprehensive database currently available for FD patients is FOS, encompassing baseline data from over 750 male and female patients with a mean age of 35 years, representing 11 countries [28,187]. Analysis of the FOS database reveals that approximately 13% of female patients (mean age, 50 years) experience cerebrovascular events (stroke or transient ischemic attacks), highlighting the importance of central nervous system (CNS) involvement in the natural history of the disease [28]. As of March 2005, 14% of females in the FOS database, with available data, reported using antidepressant medication, and 2% disclosed having made a suicide attempt [183]. Cerebrovascular lesions are identified in FD patients based on their age and the duration of disease progression. Especially in the early stages of the disease, seen in younger male and female patients, small vessels in the white matter of the brain appear to be significantly affected [188,189]. This vasculopathy, along with elevating the risk of cerebral ischemia and stroke, is likely the pathophysiological basis for cognitive impairments in FD patients. Nevertheless, there is currently insufficient evidence indicating a direct association between neuropsychological findings and alterations in morphology in the CNS of FD patients as determined by brain imaging techniques such as magnetic resonance imaging [183].

For women with FD, dealing with chronic pain and exhaustion without an adequate explanation can be quite difficult, leaving them with emotions of anger and misunderstandings. In addition, heterozygous female FD patients may experience guilt for transmitting the disorder to their offspring [23]. In a research study on four FD-affected Canadian women, all four expressed guilt of passing the disease on to their sons [184]. Emotional experiences are intimately related to psychological well-being. According to research by Fredrickson and Joiner et al., feelings of well-being have been proven to spiral upward [190]. However, there is little research on how the FD affects women psychologically. To better understand the unique psychological consequences and coping techniques of women with FD, more research is required. A multidisciplinary approach is necessary for the diagnosis and treatment of FD patients [21,148]. Healthcare providers should be aware of the disease’s possible psychological effects and offer the proper resources and assistance to support women in overcoming their obstacles.

Children with Fabry disease commonly experience symptoms such as pain, gastrointestinal dysfunction, hypohidrosis, and abnormal heat and cold tolerance [191,192]. A study on 352 patients from the Fabry Registry focused on characterizing signs and symptoms during childhood and adolescence. At enrolment, 51% of pediatric females reported symptoms, with a median onset age of 9 years. The predominant symptom in females was neuropathic pain (41%), followed by gastrointestinal symptoms (18%). Although the study didn’t measure symptom severity [193], light and electron microscopy studies of renal biopsies of nine symptomatic males and females aged 7 to 18 revealed podocyte inclusions, segmental foot process effacement, and distal tubular inclusion in all patients with normal eGFR. Albuminuria was present in 5 patients, confirming the early onset of globotriaosylceramide deposition in childhood before apparent renal disease [194]. Additionally, hearing impairment and tinnitus have been documented in children with Fabry disease [142]. Thus, many female pediatric patients with FD report early symptoms, particularly pain, gastrointestinal issues, and a diminished QoL representing an immediate burden.

## 8. Conclusions

In conclusion, FD has been considered to primarily affect males, while females were thought to be asymptomatic carriers. However, recent studies have illustrated that heterozygous Fabry women with one mutated copy of *GLA* also present an array of significant clinical symptoms that could develop into a mild to severe stage of disease. It is important to note that there is significant phenotypic variation among women with FD, likely due to random XCI. The burden of disease in women with FD is not well understood due to the low prevalence of the condition, but studies have shown that women can experience a range of symptoms, including renal manifestations. Progressive deterioration of renal function results in ERSD in some heterozygotes. Early and accurate diagnosis remains challenging due to rarity and variable presentation but advances in genetic testing and understanding molecular mechanisms may improve early and accurate diagnosis. ERT has demonstrated significant benefits in managing some of the disease’s manifestations, but its effectiveness can vary among individuals due to factors such as age of initiation, disease burden, and residual enzyme activity. The multifaceted nature of FD extends beyond its physical symptoms, encompassing emotional, psychological, and social dimensions. The physical, emotional, and psychosocial aspects converge to create a distinctive experience that requires a holistic approach from healthcare providers, researchers, and support networks. Ongoing research into FD’s pathophysiology is expected to enhance outcomes for affected women.

## Figures and Tables

**Table 1:** Biomarkers present in FD women.

Biomarker Category	Biomarkers in Women	References
Plasma	Globotriaosylceramide (Gb3)	[67]
	Lyso-Gb3	[62,72]
Urinary	Urinary globotriaosylceramide (Gb3)	[67,73]
	Urinary lyso-Gb3	[62,73]
	Urinary CDH of Ga2 (long chains isoforms)	[74]
Enzyme activity	α-Galactosidase A/LysoGb3 ratio	[66]
	α-GalactosidaseA enzyme activity	[75,76,77,78]
Genetic	Genetic mutations in the *GLA* gene	[79,80,81]
Kidney	eGFR, Creatinine, albuminuria, and proteinuria	[82,83]
Cardiac related	Troponins	[65]
	TNF, TNFR1, TNFR2 and IL6	[65,84]
	galectin-3,NT-proBNP, BNP, MRproANP, MMP2 and MMP9	[65,85]
	Detection of Fabry cardiomyopathy by CMR with LE imaging to assess LV hypertrophy and replacement fibrosis	[86]
MicroRNA profiles	Dysregulation of miRNAs associated with Fabry disease, for example, miR21-5p and miR19a-3p in the TGF-β signaling pathways	[87]

CDH: Ceramide dihexoside; Ga2: Galabiosylceramide; TNF: Tumor necrosis factor; TNFR: Tumor necrosis factor receptor; IL: Interleukin; NT-proBNP: N-terminal pro-B-type natriuretic peptide; BNP: B-type natriuretic peptide; MRproANP: mid-regional pro-atrial natriuretic peptide; MMP: matrix metalloproteinases; CMR with LE imaging: cardiac magnetic resonance (CMR) imaging–guided late enhancement (LE); LV: left ventricular; MiRNA: microRNA; TGF-β: Transforming growth factor-β.

## Data Availability

Not applicable.
